# Properties of internalization factors contributing to the uptake of extracellular DNA into tumor-initiating stem cells of mouse Krebs-2 cell line

**DOI:** 10.1186/s13287-016-0338-8

**Published:** 2016-05-25

**Authors:** Evgeniya V. Dolgova, Ekaterina A. Potter, Anastasiya S. Proskurina, Alexandra M. Minkevich, Elena R. Chernych, Alexandr A. Ostanin, Yaroslav R. Efremov, Sergey I. Bayborodin, Valeriy P. Nikolin, Nelly A. Popova, Nikolay A. Kolchanov, Sergey S. Bogachev

**Affiliations:** Institute of Cytology and Genetics, Siberian Branch of the Russian Academy of Sciences, 10 Lavrentieva Ave., Novosibirsk, 630090 Russia; Institute of Clinical Immunology, Siberian Branch of the Russian Academy of Medical Sciences, 14 Yadrintsevskaya Street, Novosibirsk, 630099 Russia; Novosibirsk State University, 2 Pirogova Street, Novosibirsk, 630090 Russia

**Keywords:** Ascites Krebs-2, Extracellular DNA, DNA internalization factors, Tumor-initiating stem cells

## Abstract

**Background:**

Previously, we demonstrated that poorly differentiated cells of various origins, including tumor-initiating stem cells present in the ascites form of mouse cancer cell line Krebs-2, are capable of naturally internalizing both linear double-stranded DNA and circular plasmid DNA.

**Methods:**

The method of co-incubating Krebs-2 cells with extracellular plasmid DNA (pUC19) or TAMRA-5’-dUTP-labeled polymerase chain reaction (PCR) product was used. It was found that internalized plasmid DNA isolated from Krebs-2 can be transformed into competent *Escherichia coli* cells. Thus, the internalization processes taking place in the Krebs-2 cell subpopulation have been analyzed and compared, as assayed by *E. coli* colony formation assay (plasmid DNA) and cytofluorescence (TAMRA-DNA).

**Results:**

We showed that extracellular DNA both in the form of plasmid DNA and a PCR product is internalized by the same subpopulation of Krebs-2 cells. We found that the saturation threshold for Krebs-2 ascites cells is 0.5 μg DNA/10^6^ cells. Supercoiled plasmid DNA, human high-molecular weight DNA, and 500 bp PCR fragments are internalized into the Krebs-2 tumor-initiating stem cells via distinct, non-competing internalization pathways. Under our experimental conditions, each cell may harbor 340–2600 copies of intact plasmid material, or up to 3.097 ± 0.044×10^6^ plasmid copies (intact or not), as detected by quantitative PCR.

**Conclusion:**

The internalization dynamics of extracellular DNA, copy number of the plasmids taken up by the cells, and competition between different types of double-stranded DNA upon internalization into tumor-initiating stem cells of mouse ascites Krebs-2 have been comprehensively analyzed. Investigation of the extracellular DNA internalization into tumor-initiating stem cells is an important part of understanding their properties and possible destruction mechanisms. For example, a TAMRA-labeled DNA probe may serve as an instrument to develop a target for the therapy of cancer, aiming at elimination of tumor stem cells, as well as developing a straightforward test system for the quantification of poorly differentiated cells, including tumor-initiating stem cells, in the bulk tumor sample (biopsy or surgery specimen).

## Background

Studies of DNA internalization by eukaryotic cells has become increasingly popular. It is well known that extracellular DNA (eDNA) can be directed into cells using transfection agents, such as lipofectamine and polyplexes [20, 37, 39]. However, eDNA has also been reported to become directly delivered to cells without the use of additional transfection tools [5, 11, 12, 14, 19, 34, 40]. Furthermore, upon internalization, eDNA sequences may become expressed [6, 14, 19].

Neither the exact mechanisms of how eDNA becomes internalized, nor the factors aiding in this process, have been comprehensively characterized. One mechanism established to mediate internalization of eDNA is endocytosis [2, 22]. In this scenario, eDNA molecules interact either with cell membrane components (adsorption endocytosis) or with appropriate cell receptors (receptor-mediated endocytosis) [28]. Experimental data are available showing that DNA molecules are transported into lymphocytes, monocytes, neutrophils, and skeletal myocytes via interaction with cell surface proteins [4, 15, 18, 35, 40]. However, most of these DNA-binding cell surface proteins have received surprisingly little attention and so their exact role in the internalization process still remains obscure. It has also been demonstrated that short nucleic acid fragments can penetrate the cell interior using special channels [7, 17, 24, 30, 36].

Our earlier studies ([12, 33] and Krebs-2 transcriptome analysis (data not shown)) indicate that Krebs-2 cells capable of internalizing eDNA (TAMRA+ cells) display properties of tumor-initiating cancer stem cells. Notably, this property is also shared by clonogenic glioma [12], multiple myeloma, and lymphoma cells (data not shown).

Both linear double-stranded DNA (dsDNA) and supercoiled plasmid DNA were shown to be taken up by Krebs-2 cells [12]. Whether these DNA species may compete with each other during internalization, and which mechanisms and factors are involved, is the focus of our research.

In the present paper, we describe internalization of pUC19 plasmid DNA by ascites cells of mouse cell line Krebs-2. We explore the efficiency of plasmid DNA internalization depending on its concentration in the medium and the incubation time. Next, we estimate whether various DNA species may competitively influence the internalization of each other (human dsDNA (0.3–6 kb) versus polymerase chain reaction (PCR) fragment (0.5 kb) versus supercoiled plasmid DNA) upon co-incubation with Krebs-2 ascites cells.

## Methods

### Laboratory animals and tumor model

We used 2- to 3-month-old CBA/Lac mice bred in the animal facility at the Institute of Cytology and Genetics, Siberian Branch of the Russian Academy of Sciences. Animals were grown in groups of 5–10 mice per cage with free access to food and water. The Ascites form of the mouse carcinoma Krebs-2 (derived from the solid form) was used as a model [21]. This cancer cell line was obtained from the cell depository of the Institute of Cytology and Genetics (Novosibirsk, Russia) and is maintained in mice as a transplanted tumor. To obtain ascites, Krebs-2 cells were diluted 1:10 in 200 μl normal saline and inoculated intraperitoneally (2 × 10^6^ cells).

### TAMRA labeling of human Alu repeat DNA and incubation of extracellular DNA with ascites Krebs-2 cells

DNA was labeled with TAMRA-5’-dUTP (N-90100, Biosan, Novosibirsk) using PCR. The PCR template was human *Alu* repeat material cloned in pBlueScript SK(+) (Alu-pBS), this repeat encompassing the tandemly repeated AluJ and AluY sequences (NCBI: AC002400.1, 53494–53767). Standard M13 primers were used for amplification. PCR purification was done by standard phenol-chloroform extraction followed by ethanol precipitation using ammonium acetate as a salt. The quantity of eDNA being added to the cells (*Alu*-TAMRA DNA, pUC19 (#440060, Medigen, Novosibirsk), pEGFP-N1 (#6085-1, Clontech), sonicated pEGFP-N1) was 1 μg plasmid DNA/10^6^ cells and 0.2 μg *Alu*-TAMRA DNA/10^6^ cells. The cells that incorporated the fluorescently labeled DNA probe were analyzed by either FACS (BD FACSAria, Becton Dickinson) or by fluorescence microscopy (laser scanning microscope LSM 510 META (Zeiss), ZEN software or AxioImager ZI microscope (Zeiss), ISIS software).

### Saturation of Krebs-2 cells with pUC19 plasmid DNA and estimates of the copy number of internalized pUC19

One million Krebs-2 cells were incubated for 1 h with pUC19 plasmid DNA (0.01; 0.1; 1; 5; 10; 20 μg). Next, to eliminate non-internalized DNA, the cells were treated with DNaseI (#18525, Serva) (10 μg/ml, 37 °С, 1 h), washed once with RPMI-1640 medium (#1.3.4, Biolot, St. Petersburg, Russia), and resuspended in 50 mM EDTA. SDS was added to a final concentration of 1 % and proteinase K (BIO-405010, Bioron GmbH, Germany) treatment (100 μg/ml) was performed at 58 °С for 1 h. Cell lysate was subjected to phenol-chloroform extraction, and the DNA was re-precipitated with 0.6 volumes of isopropanol, washed with 70 % ethanol and dissolved in water (15–40 μl). The DNA thus obtained was transformed into chemically competent XL1Blue MRF' *E. coli* cells. The cells were spread on agar-Amp plates. Colonies were counted, and this information was used to estimate plasmid copy number per cell. To verify that the transformed cells indeed carried the intended pUC19 plasmid, several individual colonies were grown in LB-Amp overnight. Plasmid DNA was purified and its identity was confirmed by gel electrophoresis.

#### Plasmid copy number estimate

The following input data were available to us: 1) *E. coli* transformation efficiency (transformation of 10 pg pUC19 plasmid DNA produced 200 colonies upon transformation); 2) 10 pg of pUC19 plasmid (2.9 kb) translates into 4.6 × 10^6^ plasmid copies; 3) the number of colonies formed upon transformation of DNA isolated from Krebs-2 cells incubated with pUC19; 4) the percentage of DNA-internalizing cells among all Krebs-2 cells is 3 % on average. Thereby, we can estimate how many cells in fact internalize DNA—3 % of 1 million cells equals 3 × 10^4^ cells,

Based on the proportion between 200 colonies and 4.6 × 10^6^ plasmid molecules, and the known number of colonies obtained in the experimental point (N), one can estimate how many plasmid molecules were present (X). Therefore, each cell internalized on average X/3 × 10^4^ plasmid molecules.

### Analysis of co-internalization of pUC19 and Alu-TAMRA DNA by Krebs-2 ascites cells

The cells were incubated with a mixture of 1 μg pUC19 and 0.2 μg *Alu*-TAMRA DNA (per one million Krebs-2 cells). Following a single wash, TAMRA+ cells were sorted using BD FACSAria flow cytometer (Becton Dickinson). Flow-sorted cells were subjected to all the treatments (starting from the DNaseI step) described in the above section of the Methods.

### Analysis of competition between different types of eDNA

*Alu*-TAMRA DNA, pUC19 (see “TAMRA labeling of human Alu repeat DNA and incubation of extracellular DNA with ascites Krebs-2 cells” section above), and human dsDNA (300–6000 bp) were used. Human DNA was isolated from placentas of healthy women using a phenol-free method, and sonicated as described in [1].

Krebs-2 cells were incubated for 1 h with the first type of eDNA, according to the above protocol. Next the cells were washed once with RPMI-1640 and resuspended in this medium. The second type of DNA was either added immediately, or the cells were left at 37 °C in the medium supplemented with 10 % fetal bovine serum (FBS; SH30071.03, HyClone, USA) until the second type of DNA was added. After the incubation with the second type of eDNA, Krebs-2 cells were washed with medium and subjected to all the procedures described in the “TAMRA labeling of human Alu repeat DNA and incubation of extracellular DNA with ascites Krebs-2 cells” section above.

### Cell cycle analysis of TAMRA+ and unsorted Krebs-2 cells

Sorted TAMRA+ or unsorted Krebs-2 cells were centrifuged at 400 g, 4 °С for 5 min, and fixed in 60 % methanol for 1 h at 4 °С. The cell suspension was pelleted, washed with phosphate-buffered saline (PBS), and treated with 200 μg/ml RNase (LLC Samson-Med, St. Petersburg, Russia) for 30 min at 37 °С. Next, propidium iodide (P4170, Sigma-Aldrich) was added to the cell suspension for 10 min at room temperature. Cell cycle profiling was performed using BD FACSAria flow cytometer (Becton Dickinson).

### Quantitative PCR quantification of eDNA copy number in Krebs-2 cells

#### Isolation of DNA

Following incubation of Krebs-2 cells with eDNA and DNaseI treatment, the cell membrane was lysed with 0.5 % Triton-X100 (#37238, Serva) (15 min on ice). Nuclei were pelleted by centrifugation at 200 g for 5 min at 4 °C. Supernatants were collected and DNA was precipitated by adding 0.6 volumes of isopropanol. The pellets were re-dissolved in a small volume of water. Nuclear pellets were resuspended in 50 mM EDTA, SDS was added to 1 %, and samples were treated with proteinase K. DNA was purified by phenol-chloroform extraction and re-precipitated as described above.

#### Quantitative PCR and calibration curve

DNA molecules were quantified by real-time PCR using SYBR Green PCR Master Mix (#4309155, Applied Biosystems, UK). To generate the quantitative PCR (qPCR) calibration curve, standard M13 primers or primers from the Amp gene (forward: 5’-ATGAGTATTCAACATTTCCG-3’; reverse: 5’-GATCTTACCGCTGTTGAGAT-3’) were used, and 0, 0.5, 5, 50, 500, 5000 and 50,000 pg of each pUC19 and *Alu-*repeat DNA were added to the reactions. Each concentration was run in triplicate. The linear fit of Ct versus eDNA content was plotted using StepOne Software v2.3. pUC19 and *Alu* DNA present in the nuclear or cytoplasmic fractions of Krebs-2 cells was quantified using StepOne Software v2.3. Template DNA (100 ng) was added to each qPCR reaction. DNA isolated from intact Krebs-2 cells was used as a negative control (and no product whatsoever was observed). All real-time PCR experiments were performed in triplicate and repeated twice on a Step One Real-Time PCR System (Applied Biosystems).

#### Conversion of qPCR data into eDNA copy numbers

Calibration curve-based qPCR data were converted into absolute plasmid or *Alu*-repeat molecule numbers as follows: 100 ng of Krebs-2 DNA added to each qPCR equals ~8333 cells (12 pg/cell). Given than TAMRA+ cells were shown to be the same cells as those internalizing plasmid DNA, we could estimate the exact percentage of cells that internalized both types of eDNA. From fluorescence microscopy analysis, we know that 2 % of cells were eDNA-internalizing (i.e., ~167 cells). Hence, by dividing the eDNA copy number measured by 167, one obtains the number of eDNA molecules per cell.

### Statistical analysis

Statistical analysis was performed using Statistica 10 software. In the figures, bars show standard deviation (*n* = 3–4 at different experimental points). The level of significance was estimated using Students *t* tests.

## Results

### Internalization of Alu-TAMRA dsDNA and supercoiled plasmid pUC19 DNA by Krebs-2 cells

**Fig. 1:**
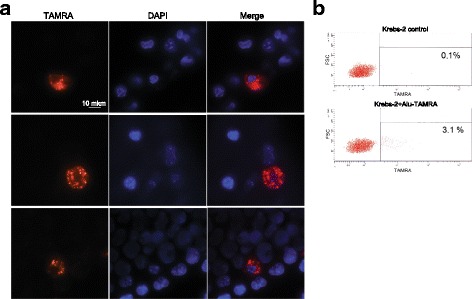
Cytofluorescence (**a**) and flow cytometry (**b**) analyses of *Alu*-TAMRA DNA internalization by ascites Krebs-2 cells. DNA is stained with DAPI (*blue*); TAMRA (*red*) corresponds to *Alu*-TAMRA DNA

Previously, passaging the ascites in a grafted form was demonstrated not to affect the ability of a subpopulation of ascites cells (tumor-initiating stem cells (TISCs)) to internalize extracellular dsDNA in the absence of additional transfection factors [12] (Fig. 1). The percentage of Krebs-2 cells that internalized *Alu*-TAMRA DNA has been analyzed by confocal microscopy and cytometry and was within the previously reported experimental range (1–7 %), namely 3 %. Performing the incubation of Krebs-2 cells with *Alu*-TAMRA DNA at 4 °С, 25 °С, or 37 °С did not influence the efficiency of internalization.

**Fig. 2:**
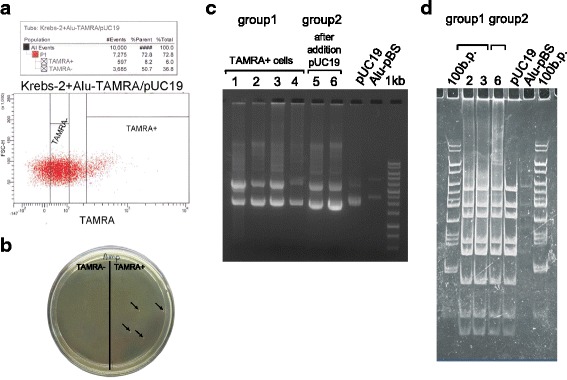
Analysis of plasmids isolated from the colonies obtained by transformation of competent *E. coli* cells with DNA from Krebs-2 ascites pre-incubated with different types of eDNA (pUC19 only or pUC19 + *Alu*-TAMRA dsDNA). **a** Krebs-2 ascites cells were incubated with a mixture of plasmid pUC19 DNA and linear *Alu*-TAMRA dsDNA. TAMRA+ and TAMRA– subpopulations were gated as shown. **b** Image of colony growth on an LB-amp plate seeded with *E. coli* cells transformed with DNA from TAMRA+ or TAMRA– Krebs-2 subpopulations. No colonies are formed in the latter group. **c** Agarose gel electrophoresis analysis of the plasmids recovered. 1–4, Plasmids obtained from TAMRA+ material; 5, 6, plasmids obtained from the control transformation (Krebs-2 cells incubated with pUC19 DNA only); pUC19, Alu-pBS, original plasmids; 1 kb, DNA molecular weight 1 kb ladder. **d** Restriction analysis of plasmid DNA with a 4-cutter *Hae*III. Plasmids derived from TAMRA+ cells (2, 3) correspond to pUC19, much as the plasmid (6) isolated from control Krebs-2 cells incubated with just pUC19

First and foremost, we wanted to understand whether Krebs-2 cells internalizing linear *Alu*-TAMRA DNA are the same cells that internalize supercoiled plasmid DNA. To do this, ascites Krebs-2 cells were co-incubated with TAMRA-labeled *Alu* DNA and supercoiled pUC19 plasmid DNA. The cells were flow-sorted into TAMRA-positive and -negative subpopulations. Their DNA was isolated and transformed into competent *E. coli* cells. Upon transformation, only TAMRA+ cells produced *E. coli* colonies. Plasmid DNA isolated from these colonies was identical to the original pUC19 plasmid, which was used for co-incubation experiments (Fig. 2).

### Copy number analysis of dsDNA molecules internalized by Krebs-2 TISCs

**Fig. 3:**
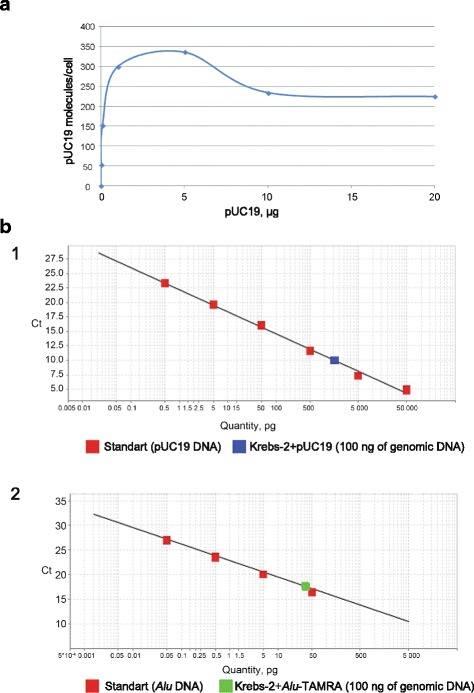
Copy number analysis of eDNA internalized by Krebs-2 cells. **a** saturation of Krebs-2 cells with increasing amounts of pUC19 DNA in the incubation medium. **b** qPCR quantification of pUC19 (1) and *Alu* DNA (2) present in Krebs-2 cells after co-incubation with eDNA. Linear calibration plot was constructed using StepOne v2.3 software; each datapoint was run in triplicate

Selective targeting of TISCs by delivery of cell-killing genes carried on the plasmids is an attractive approach with a clear therapeutic application. For this approach to work, it is important to know whether non-degraded DNA molecules are delivered to the cells and how many native plasmid DNA molecules can be found in the cell at a time. To address these questions, two series of experiments were carried out. In the first series, we estimated the number of plasmid DNA molecules internalized by Krebs-2 cells by transformation of DNA from such cells into competent *E. coli*. Also, using an *E. coli* transformation assay, we determined the saturation threshold of Krebs-2 cells, which is 1 μg/10^6^ cells (Fig. 3a). In the second series, the internalized plasmid DNA was quantified directly using qPCR. Importantly, unlike transformation, qPCR quantifies the entire pool of DNA molecules, regardless of whether they are nicked, partially degraded, or integrated into the genome or not. The results expectedly differ by three orders of magnitude: transformation experiments give an estimate from 340 (Fig. 3a) to 2600 (data not shown) plasmid copies/cell, with qPCR producing an estimate of 1.070 ± 0.054×10^6^ copies/cell and 3.097 ± 0.044×10^6^ copies/cell (Amp and M13 primer sets, respectively) (see Methods) (Fig. 3b-1). We also analyzed the copy number of *Alu* fragments internalized by the cells, which was 0.425 ± 0.011×10^6^ (Fig. 3b-2). Additionally, we monitored the dynamics of *Alu-*TAMRA internalization by Krebs-2 TISCs. The cells become saturated with eDNA fragments by the end of the first hour of incubation. According to the confocal imaging analysis (data not shown), internalized labeled DNA material appears to double every 10 min.

### Competitive interactions between human dsDNA, yeast RNA, bovine serum albumin, and heparin during internalization of pUC19 by Krebs-2 cells

**Fig. 4:**
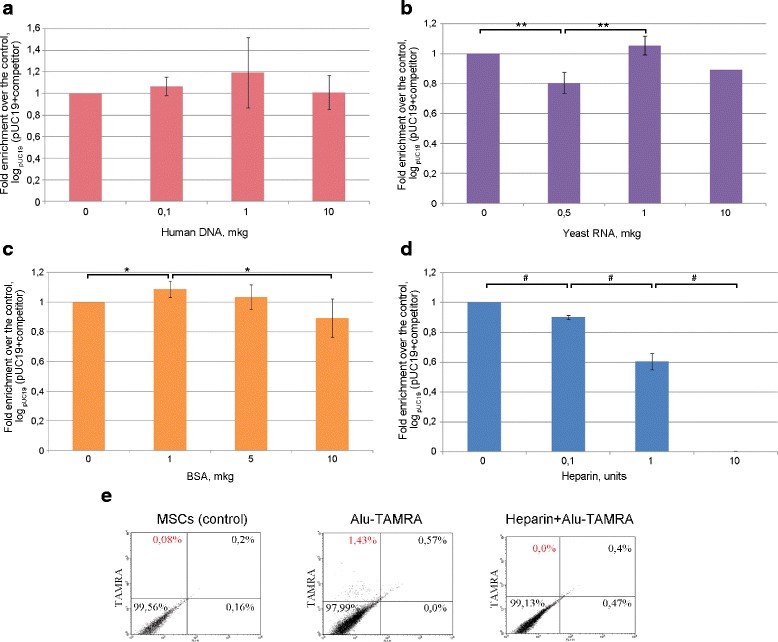
Internalization of supercoiled pUC19 plasmid DNA by Krebs-2 cells in the presence of various competitors. The relative internalization efficiency of pUC19 plasmid DNA in the presence of different amounts of human dsDNA (**a**), yeast RNA (**b**), bovine serum albumin (*BSA*) (**c**), and heparin (**d**). The values are shown on a log _pUC19_ (pUC19 + competitor) scale. **e** FACS analysis of *Alu*-TAMRA dsDNA internalization by human mesenchymal stem cells (*MSCs*) in the presence or absence of heparin. The data obtained suggest that heparin is a dose-dependent inhibitor of plasmid DNA internalization and suppresses linear DNA internalization by the cells. *p < 0.05; **p < 0.01; ^#^ *p* < 0.001

Next, we proceeded to characterize possible competition between supercoiled plasmid DNA (pUC19) and linear DNA (fragmented human genomic DNA), total yeast RNA, bovine serum albumin (BSA), and heparin. Neither linear dsDNA (300–6000 bp), nor RNA, nor BSA compete with plasmid DNA for internalization factors (Fig. 4a, b and c). Amazingly, the addition of 10 U heparin (7.7 μg) abolished the internalization by Krebs-2 cells (Fig. 4d). In parallel, heparin was also demonstrated to abolish the uptake of *Alu*-TAMRA dsDNA by human mesenchymal stem cells (Fig. 4e).

### Competition between various eDNA types during internalization by Krebs-2 cells

**Fig. 5:**
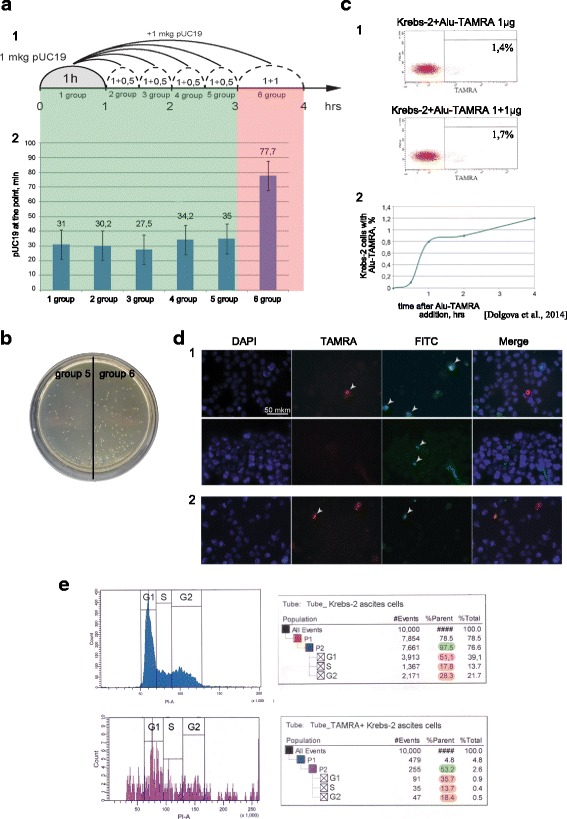
Internalization of eDNA by Krebs-2 cells upon additional rounds of incubation. **a** Internalization of pUC19 plasmid was analyzed when this plasmid was added every 30 min (groups 2–6) following 1-h pre-incubation with 1 μg pUC19 (group 1). Shown is the total number of colonies obtained by transforming competent *E. coli* with DNA from 10^6^ Krebs-2 cells incubated with pUC19 DNA; **b** representative image of *E. coli* colonies that formed as a result of transformation of DNA isolated from Krebs-2 cells that received additional pUC19 DNA 2.5 (group 5) and 3 h (group 6) after the pre-incubation step. Many more colonies are visible on the group 6 half of the plate; **c** FACS analysis of *Alu*-TAMRA dsDNA internalization by Krebs-2 cells 3 h following the pre-incubation with the same DNA; **d** fluorescence microscopy analysis of Krebs-2 cells pre-incubated with *Alu*-TAMRA DNA, followed by addition of *Alu*-FITC DNA. Cells internalizing fluorescent probes are marked with *arrows*; **e** cell cycle profiling of the total (unsorted) cell population (*top*), and TAMRA+ Krebs-2 cells (*bottom*). Percentages of cells found in G1, S, and G2/M phases are shaded in *red*

**Fig. 6:**
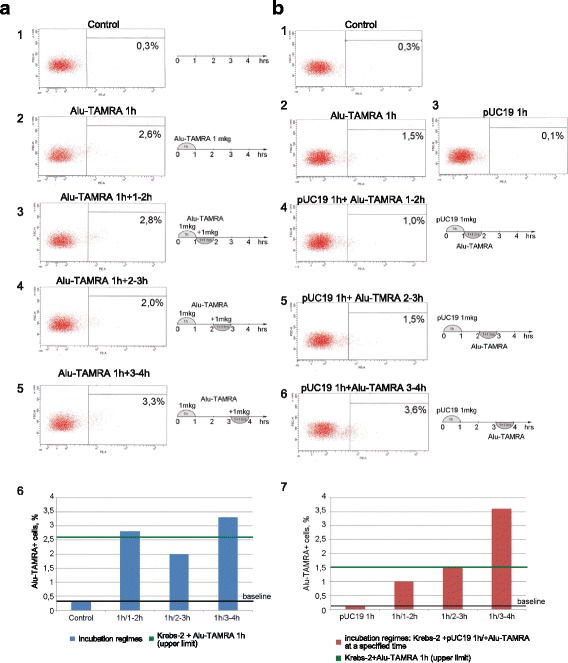
Competition between *Alu*-TAMRA dsDNA and supercoiled pUC19 plasmid DNA for internalization into Krebs-2 cells. **a** *Alu*-TAMRA DNA is added 1 h after pre-incubation with the same DNA. **b** *Alu*-TAMRA DNA is added after pre-incubation with pUC19. 1a-5a, 1b-6b, Flow cytometry plots and schematics of each experimental point analyzed; 6a, 7b, percentage of TAMRA+ cells analyzed at each point. Baseline equals background level of fluorescence, i.e., either when no DNA was added (**a**), or when cells were incubated for 1 h with non-fluorescent plasmid DNA (**b**); upper limit represents percentage of TAMRA+ cells following incubation with *Alu*-TAMRA DNA

**Fig. 7:**
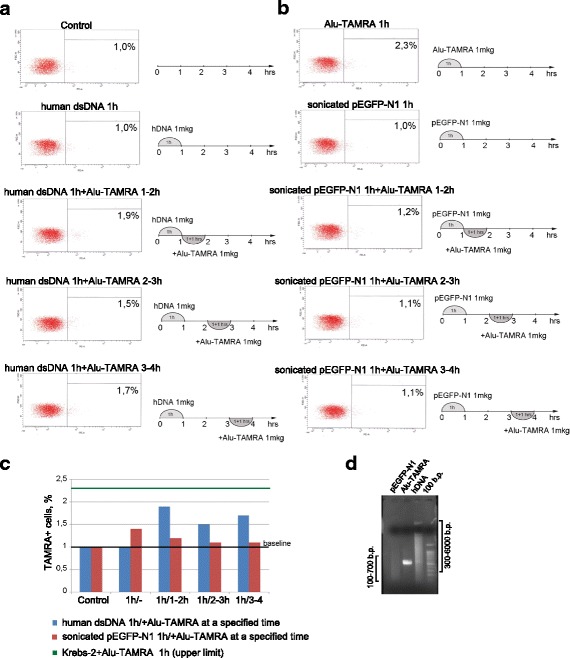
Competition between *Alu*-TAMRA DNA and high-molecular weight human DNA or sonicated pEGFP-N1 plasmid for internalization into Krebs-2 cells. **a** Addition of *Alu*-TAMRA DNA to Krebs-2 cells pre-incubated with human dsDNA for 1 h; **b** addition of *Alu*-TAMRA DNA to Krebs-2 cells pre-incubated sonicated pEGFP-N1 plasmid DNA. FACS plots and schematics of the experimental points analyzed are shown; **c** bar plot summarizing the percentages of TAMRA+ cells. Baseline equals background fluorescence of untreated cells; control represents cells incubated for 1 h with 1 μg of either human dsDNA (*blue*) or sonicated pEGFP-N1 plasmid (*red*); upper limit represents percentage of TAMRA+ cells after 1 h incubation with 1 μg *Alu*-TAMRA DNA; **d** results of an agarose gel electrophoresis showing fragment size distributions of the three eDNAs tested. Brackets on the sides of the gel indicate average sizes of sonicated pEGFP-N1 (*left*) and human dsDNA (*right*) fragments. *dsDNA* double-stranded DNA

Our earlier studies showed that internalization of extracellular DNA by human MCF-7 cells proceeds in two waves, and peaks at 1 and 3 h after the beginning of incubation [34]. In the present work, a series of experiments was performed to characterize the dynamics of eDNA internalization when Krebs-2 cells have been pre-incubated with various types of dsDNA. Specifically, one million Krebs-2 cells were incubated for 1 h with different dsDNA preparations. Next, additional DNA samples were added to the cells (once every hour, 3–4 h in total). To eliminate non-internalized DNA (unbound or membrane-associated DNA), the cells were finally treated with DNaseI. All experiments involved either pUC19 plasmid DNA or TAMRA-labeled human *Alu* repeat. These served as references for comparisons (*E. coli* transformation assay for pUC19, and flow cytometry for *Alu*-TAMRA). Several types of competition experiments were performed; each was run several times to achieve statistical significance. The following combinations were tested: pUC19 + pUC19 (Fig. 5), *Alu*-TAMRA + *Alu*-TAMRA (Fig. 6a), pUC19 + *Alu*-TAMRA (Fig. 6b), total human DNA + *Alu*-TAMRA (Fig. 7a), and sonicated pEGFP-N1 + *Alu*-TAMRA (Fig. 7b). The reasons for choosing these specific DNA combinations are explained below.

**Fig. 8:**
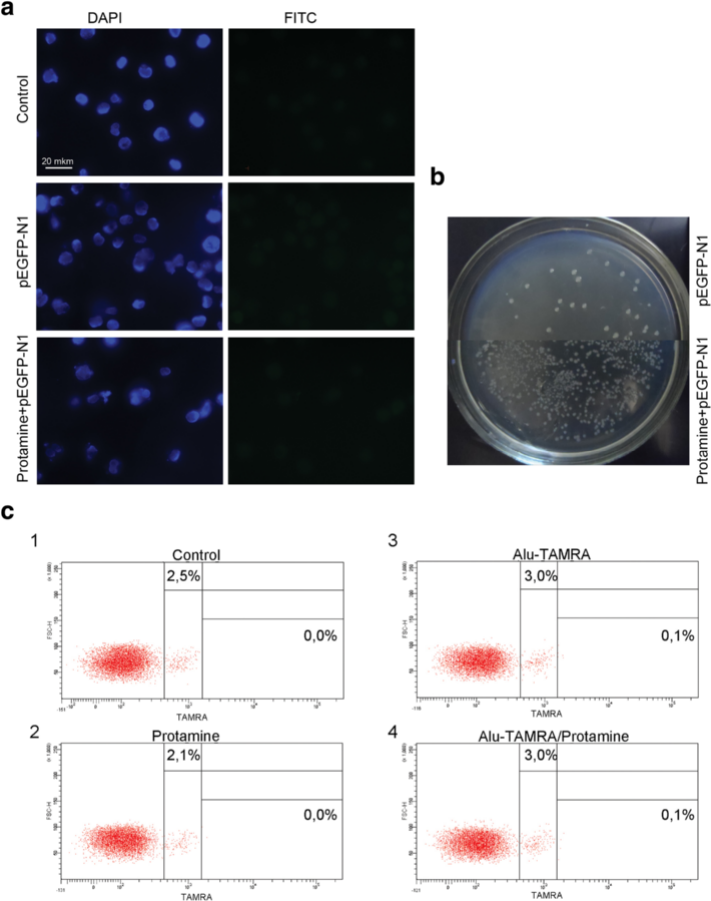
Internalization of protamine/extracellular DNA complexes by Krebs-2 cells. **a** Analysis of GFP fluorescence in Krebs-2 cells following incubation with pEGFP-N1 plasmid. DAPI represents chromatin *(blue channel*); FITC represents GFP (*green channel*, no signal). **b** Transformation of *E. coli* competent cells with DNA isolated from Krebs-2 cells incubated with pEGFP-N1 or pEGFP-N1/protamine complexes. **c** FACS analysis of *Alu*-TAMRA DNA/protamine internalization by Krebs-2 cells: 1: Negative control, intact Krebs-2 cells; 2: Krebs-2 + protamine; 3: Krebs-2 + *Alu*-TAMRA DNA; 4: Krebs-2 + *Alu*-TAMRA DNA/protamine complexes. Percentage values correspond to autofluorescence levels observed in the control and to the fraction of TAMRA+ cells at experimental points

1. *pUC19 + pUC19*
  First, we wanted to confirm or challenge the existence of a second wave of internalization 3–4 h after the beginning of incubation, as was demonstrated for MCF-7 cells (see Methods). Indeed, we observed that adding more plasmid DNA 3–4 h following the incubation results in significantly more plasmids capable of forming colonies upon transformation into competent *E. coli* (Fig. 5a and b). This is formally compatible with two basic scenarios: either more internalization-competent cells become available with time, or more plasmid copies accumulate in the same cells. To understand which scenario is correct, fluorescently labeled DNA was included in the analysis (*Alu*-TAMRA). Using flow cytometry, we show that there is a 20 % gain in the TAMRA+ cell subpopulation (from 1.4 % to 1.7 %) when *Alu*-TAMRA DNA is added 3 h after beginning the first incubation (Fig. 5c-1). This observation is consistent with our earlier report [12] where the percentage of TAMRA+ cells was found to increase upon a longer incubation time (Fig. 5c-2). We performed an additional experiment wherein the cells were first incubated with *Alu*-TAMRA DNA, followed by *Alu*-FITC DNA 3 h later. The cells were analyzed under a fluorescence microscope. This analysis showed that the second portion of eDNA was largely absent from TAMRA+ cells, and is restricted to TAMRA cells (Fig. 5d-1). Cells that were double-positive for *Alu*-TAMRA and *Alu*-FITC (Fig. 5d-2) were present, but were rather rare. Taken together, these data suggest that 3–4 h following the beginning of the incubation there is a 13–50 % increase in the number of cells internalizing exogenous DNA.
  Next, we compared cell cycle distributions of unsorted cells and eDNA-internalizing cells (Fig. 5e). The two profiles were indistinguishable, with about 20 % of cells found in the G2-M phases. One can speculate that the cells undergoing mitosis are unlikely candidates for a cell-internalizing subpopulation. However, upon exiting the M phase such cells may become internalization-competent. The timing of the second wave of internalization events matches the length of the M phase, which is known to be 3–4 h. Data on the percentage of dividing cells and the increase in TAMRA+ subpopulation at a 4-h timepoint indicate that the increase in the plasmid copy number is attributable to the availability of an additional pool of internalization-competent cells that were undergoing mitosis and were incapable of internalizing DNA.
  Next, we aimed to address the question whether *Alu*-TAMRA PCR fragments and supercoiled DNA are internalized using the same factors or not. Two combinations of DNA were tested: *Alu-*TAMRA + *Alu*-TAMRA (Fig. 6a) and pUC19 + *Alu*-TAMRA (Fig. 6b). The first combination was necessary to control for the effect of the second wave of internalization. The second combination allowed estimating the contribution of both types of DNA into internalization, so as to understand whether internalization factors used for *Alu*-TAMRA and plasmid DNA are the same.
2. *Alu-TAMRA + Alu-TAMRA*
  When Krebs-2 cells are pre-incubated with *Alu*-TAMRA DNA and then additional portions of the same DNA are introduced, the pre-incubation step should saturate all the G1-S cells available and no increase in TAMRA-positive cells should be observed during the first 3–4 h. The increase in internalizing cells (about 20 %) should, however, be observed after the 3–4 h timepoint, which corresponds to the percentage of cells found in G2-M phases in the beginning of the experiment. Data obtained for *Alu*-TAMRA DNA appear similar to those obtained for the plasmid DNA, i.e., there is no internalization during the first 3 h and a second wave of internalization events follows immediately after (Fig. 6a 1–6). The plot shown in Fig. 6a indicates that the percentage of TAMRA+ cells remains unchanged at 1-h, 2-h, and 3-h timepoints (upper limit). However, it does increase some 13 % later on (from 2.6 % to 3.3 %) (Fig. 6a 2,5,6).
3. *pUC19 + Alu-TAMRA*
  Should the factor controlling internalization of *Alu*-TAMRA and plasmid DNA be the same, pre-incubation of Krebs-2 cells with plasmid DNA should prevent TAMRA+ cells from appearing following the incubation steps, and thus internalization above the “upper limit” should only become detectable 3–4 h after the beginning of the experiment. Alternatively, if internalization factors are different, incubation with *Alu*-TAMRA DNA should immediately result in a TAMRA+ cell subpopulation, with the percentage matching that of the control (*Alu*-TAMRA, 1 h).
  What we observed when testing this combination was that, following pre-incubation with pUC19, the percentage of *Alu*-TAMRA cells equaled the control level (as in *Alu*-TAMRA 1 h). After 4 h, the second wave of internalization occurs. No decrease in the percentage of *Alu*-TAMRA-internalizing cells was observed, when they were saturated by the pre-incubation with plasmid DNA (Fig. 6b 2,4,5). These results indicate that Krebs-2 cells use at least two distinct internalization pathways for the uptake of supercoiled plasmid DNA and linear TAMRA-labeled *Alu* DNA (500 bp) (Fig. 6b). The conclusions of these experiments are in line with co-incubation experiments using plasmid DNA and human dsDNA (300–6000 bp) showing lack of competition between the two species for internalization factors (Fig. 4a).
  The final experimental series was performed to estimate the competition between various types of linear DNA. Two combinations were selected: human DNA + *Alu*-TAMRA (Fig. 7a), and sonicated pEGFP-N1 + *Alu*-TAMRA (Fig. 7b), and analyzed as above.
4. *human DNA+ Alu-TAMRA*
  Testing this combination helped address possible competition with human genomic DNA sonicated to the size of 300–6000 bp (as this DNA species was previously demonstrated to internalize into Krebs-2 TISCs [12]). The results obtained show that internalization factors for 300–6000 bp human DNA (hereafter referred to as high-molecular weight DNA) and human *Alu* repeat provided as a PCR fragment are distinct (Fig. 7a and c). We observed that there was partial competition between *Alu* DNA and sonicated human DNA for internalization factor(s), as demonstrated by the slight decrease in *Alu*-TAMRA-internalizing cells. This is likely attributable to the presence of trace amounts of DNA below 500 bp in the sonicated human DNA preparation, which serves as a competitor.
5. *Sonicated pEGFP-N1 + Alu-TAMRA*
  Use of this combination focused the analysis on the competition between DNA species similar in structure and size (Fig. 7d). Internalization of 500 bp *Alu*-TAMRA PCR product by Krebs-2 cells pre-incubated with sonicated pEGFP-N1 plasmid (<700 bp) was significantly suppressed, which suggests competition for the internalization factor (Fig. 7b and c).
  Taken together, our data suggest that supercoiled plasmid DNA reaches internal compartments of Krebs-2 TISCs essentially intact, given that functional plasmids can be recovered upon transformation into competent *E. coli* cells. Hence, it was interesting to test whether GFP could be expressed from the pEGFP-N1 plasmid internalized by Krebs-2 TISCs. Cells and the plasmid DNA were co-incubated for 1 h. Then incubation medium was replaced with a 10 % FBS-supplied medium, and cells were kept in the CO_2_ incubator for another 24 h. We ensured pEGFP-N1 plasmid was fully functional by incubating it with mouse fibroblasts. In the presence of lipofectamine, about 70 % of cells were scored GFP-positive. Two incubation protocols were tested for Krebs-2 TISCs, one including protamine, and one with protamine omitted. When plasmid DNA was pre-incubated with protamine, this resulted in a 17-fold increase in internalization efficiency. Notably, in neither protocol was any GFP fluorescence observed in Krebs-2 TISCs (Fig. 8a and b). Control experiments involving *Alu*-TAMRA DNA or *Alu*-TAMRA “pre-complexed” with protamine indicate that the percentage of DNA-internalizing cells remains unchanged (Fig. 8c). This argues for the existence of an additional internalization factor for DNA-protamine complexes.

## Discussion

In the present study, we performed comprehensive analysis of plasmid DNA internalization by Krebs-2 cells, and established several interesting aspects of competition between distinct DNA species upon internalization. To do so, we developed a special approach wherein Krebs-2 cells were first incubated or co-incubated with plasmid DNA; total DNA was isolated from such cells and used for transformation of competent *E. coli* cells*.* The rationale behind this approach is that it is important to analyze whether plasmid DNA can be used as a versatile vector to deliver cell-killing genes to TISCs. Efficiency of internalization was also assayed independently using qPCR.

About 3 % of mouse ascites Krebs-2 cells were shown to internalize eDNA, such as TAMRA-5’-dUTP human *Alu* DNA fragment (Fig. 1). Saturation of the cells by *Alu* DNA fragments was achieved during the first hour of incubation, and the quantity of internalized DNA material doubled every 10 min. The efficiency of internalization remained unaffected across a wide range of temperatures tested (4 °С, 25 °С, and 37 °С).

We demonstrate that internalization of dsDNA (500 bp) and supercoiled pUC19 plasmid DNA occurs in exactly the same tumor cells (Fig. 2). The cell saturation threshold with plasmid DNA was defined. When 1 μg plasmid DNA is co-incubated with 10^6^ Krebs-2 cells, ascites cells cannot internalize more eDNA. Such cells encompass 340–2600 copies of the plasmid, depending on the transformation experiment, or up to 3.097 ± 0.044×10^6^ molecules, as assayed by qPCR (Fig. 3). The approximate 1000-fold difference between the results obtained using these two quantification methods is explained by the distinct sensitivities of these approaches. The number of colonies obtained after transformation is affected by many parameters, such as the competence level of bacterial cells, sterical interactions of circular plasmid DNA with chromatin, association of cellular proteins with the internalized plasmids, and structural integrity of the plasmid. In contrast, these factors are of little importance when PCR quantification is used, as it only measures the number of target molecules delimited by the primer binding sites.

When eDNA is condensed by forming complexes with a positively charged protamine [9], this results in a 17-fold increase in internalization, with the percentage of DNA-internalizing cells remaining unchanged.

Experiments were performed to analyze possible competition between plasmid pUC19 and various forms of nucleic acids (human dsDNA (300–6000 bp), PCR fragment (500 bp), yeast RNA), as well as other agents (BSA, heparin) for internalization by Krebs-2 cells. Of these, only heparin worked as a potent inhibitor of internalization (Fig. 4). These observations are consistent with the idea that heparin interacts with the same internalization factor(s) as plasmid DNA. Heparin was reported to interact with a number of cell surface molecules, such as heparin binding protein and CAP37/azurocidin [16]. Bennett and colleagues showed that heparin and DNA molecules compete for binding to a 30-kDa protein in the context of lymphocyte membranes, whereas RNA, poly-dA/dT, or dNTP mix do not [3]. Also, heparin is known to interact with a family of fibroblast growth factor receptors [13]. Exosomes have been reported to contain dsDNA, and so their cargo can be easily internalized—in a process that is inhibited by heparin [8]. This could have pointed to the possible involvement of exosomes in eDNA internalization. We checked whether the TAMRA+ probe could be effluxed by Krebs-2 TISCs and observed that, upon internalization, the DNA did not leave the cells in any form, including exosomal (data not shown). This indicates that internalized DNA does not become part of exosomes, at least in the given context. The formal possibility that an exosome would internalize extracellular dsDNA appears rather unlikely, nor has such a property been reported in the literature. Given the above-described range of cell receptors bound by heparin and its property to inhibit eDNA internalization, one can expect that in the case of Krebs-2 cells these cell surface proteins are bona fide eDNA internalization factors.

Depending on the experiment, we observed increased internalization (13–50 %) of plasmid DNA 3 h after the beginning of incubation (Fig. 5). Formally, this observation is compatible with two scenarios. The first implies that internalization is mediated by specific cell receptors that shuttle back and forth between the cell surface and the cell interior [40]. The other relies on the greater availability of internalization-competent cells. We showed that TAMRA+ cells are the same cells as those internalizing plasmid DNA, and that their percentage ranges 1–3 % in our experiments. Under the first scenario, the percentage of *Alu*-TAMRA internalizing cells should remain unchanged at the 3- to 4-h timepoint; however, this was not the case, and more TAMRA+ cells were observed 3 h following the beginning of incubation (Fig. 5c). Thus, the second scenario appears the likeliest. Internalization of pUC19 translates into more colonies because more internalization-competent Krebs-2 cells become available, rather than because more plasmid copies are internalized.

Krebs-2 TISCs are not expected to internalize eDNA during mitosis, yet they become internalization-competent soon thereafter. Importantly, all other cells of this particular cell subpopulation already contain eDNA molecules. Thus, there should be a gain in DNA-internalizing cells that should be equal to the proportion of cells found in the G2-M phases in the beginning of the experiment, which in turn should translate into more *E. coli* colonies formed upon transformation. The cells that internalized DNA in G1 persist until mitosis, which explains why cell cycle profiling experiments failed to uncover significant accumulation of TAMRA+ cells in any of the cell cycle phases (Fig. 5a, c and e; [12]).

Finally, we tested competition between supercoiled plasmid DNA (pUC19), short PCR product (human *Alu* PCR fragment, 500 bp), sonicated pEGFP-N1 plasmid (100–700 bp), and larger-sized sonicated human dsDNA (300–6000 bp). If internalization factors for distinct types of DNA are the same, then saturating pre-treatment with one DNA species should block internalization of the other. Alternatively, should internalization factors be distinct, the second DNA type should be internalized regardless of the pre-treatment type of DNA. It turned out that pre-treatment with *Alu* PCR fragment abolishes subsequent internalization of *Alu* DNA and sonicated pEGFP-N1 by Krebs-2 cells (Figs. 6a and 7b). We attribute this to the fact that sonicated plasmid DNA is similar in size (100–700 bp) to *Alu* fragment (500 bp), thereby efficiently competing for binding to the receptor that mediates internalization of small (up to 500 bp) DNA fragments. The same was observed for plasmid DNA that blocked its own internalization. Efficiency of protamine-assisted eDNA internalization was also analyzed.

Pre-treatment of Krebs-2 cells with supercoiled plasmid DNA does not affect subsequent internalization of *Alu* PCR fragment (Fig. 6b). This is unlike pre-treatment with sonicated human dsDNA, which shows partial inhibition of *Alu*-TAMRA DNA internalization by Krebs-2 cells (Fig. 7a). We believe this partial inhibition is due to the presence of small amounts of low-molecular weight DNA fragments (<500 bp) in the preparation of sonicated human DNA (300–6000 bp). Most of the fragments in this preparation, however, are longer than 1 kb, and so they do not compete for binding to the internalization factor that mediates the uptake of fragments below 500 bp.

### Biological meaning and clinical significance of the phenomenon observed

The phenomenon of internalization of extracellular dsDNA by stem cells (SCs) and TISCs is broadly related to the functioning of an organism as a single entity. We speculate that continuous flow of a highly dynamic set of extracellular DNA fragments through various SCs may function as a mechanism that helps such cells sense the genetic “image” of the body. Quantitative as well as qualitative changes among these eDNA molecules are interpreted by the multipotent cells as the clues setting the direction of differentiation.

Upon internalization and reaching the internal compartments of SCs and TISCs, eDNA may participate in many molecular processes. For instance, it is known that: 1) Any free dsDNA ends, including the ends of eDNA molecules, potently induce repair cascades [23, 29, 41–43]. Our studies as well as other reports indicate that, upon internalization, linear plasmid DNA is partially digested at its termini and is ligated to form a circle [11, 25, 32, 34]. 2) When the cell is undergoing the repair of interstrand DNA crosslinks induced by an earlier treatment with a cytostatic drug, eDNA fragments delivered at this point into the cell interfere with nucleotide excision repair and homologous recombination repair phases. In the context of murine CD34+ hematopoietic stem cells (HSCs), this results in the failure to give rise to lymphoid cell lineage [10], whereas Krebs-2 TISCs either die or become non-tumorigenic [33]. 3) When the cells repairing radiation-induced dsDNA breaks receive eDNA fragments, the latter interfere with the non-homologous end-joining step, yet HSCs are rescued from the aberrant recovery of chromosomal integrity and apoptosis. These surviving HSCs help repopulate the murine hematopoietic system, thereby protecting the animals from developing radiation sickness and death, and increasing the survival of lethally irradiated mice to 60–90 % [26]. Thus, “planting” eDNA to TISCs in a timely manner, when these cells are trying to repair the DNA, may potently interfere with the repair process, and so poorly differentiated cell types including TISCs either die or profoundly alter their properties. Clearly, this approach relies on fundamentally different principles of targeting SCs and TISCs. One of the most intriguing questions then is whether extracellular fragmented dsDNA may also target solitary cancer cells that give rise to metastases and which are known to be largely quiescent (and represent a form of TISCs). The very existence of these cells prompts for the careful analysis of many interesting questions related to cancer biology and therapy [31, 38]. In the absence of experimental data, we propose several ideas on the possible interplay between solitary cancer cells and fragmented dsDNA. First, these cells may be imagined not to internalize eDNA, and it is important to understand why poorly differentiated cells otherwise classifiable as TISCs fail to do so [12]. Alternatively, dormant cancer cells do internalize dsDNA, but whether their quiescence may prevent the fragmented DNA from efficiently interfering with the aforementioned molecular processes remains unclear. A number of so-called stress signals have been reported to induce proliferation of dormant cancer cells, which leads to the development of a metastasis [38]. dsDNA fragments internalized by solitary cancer cells may be viewed as a classical stress signal. Our work, as well as the data from other research groups, is consistent with the following scenario. Free ends of internalized dsDNA molecules launch a kinase cascade thereby leading to the cell cycle arrest and block of cell division [23, 25, 29, 32, 41–43]. Our unpublished ex vivo studies indicate that dsDNA fragments are delivered into CD34+ HSCs, most of which are non-dividing, and paradoxically induce their proliferation. We speculate that stalling of the cell cycle prevents the HSC from reverting to the quiescent state, thereby forcing the dsDNA-activated cell to proliferate [27]. This scenario may point to the possibility that solitary cancer cells may be induced to proliferate by extracellular DNA fragments, which should translate into a clinically negative effect. Nevertheless, if dormant cancer cells do internalize eDNA this may well be used as a tool to selectively kill such cells, essentially in the same way as recently proposed [33]. Thus, our approach primarily targets poorly differentiated cell types including TISCs. Expression of a toxic protein or other protein of interest delivered to such cells in the form of DNA may be used to selectively alter their phenotype or eliminate them altogether.

## Conclusion

Upon reaching the cell interior, eDNA molecules do not behave as an inert cargo. They are actively integrated in multiple on-going cellular processes and frequently compromise their correct progression. Additionally, extracellular DNA may also induce a number of cellular processes. Finally, the very property of dsDNA internalization may serve to develop a versatile approach to detect and target any type of poorly differentiated cells, such as TISCs.

## Abbreviations

Alu-TAMRA, human Alu repeat DNA labeled by the fluorescently modified nucleotide TAMRA-5’-dUTP; BSA, bovine serum albumin; dsDNA, double-stranded DNA; eDNA, extracellular DNA; FBS, fetal bovine serum; HSC, hematopoietic stem cell; PCR, polymerase chain reaction; qPCR, quantitative polymerase chain reaction; SC, stem cell; TISC, tumor-initiating stem cell.
